# L-Type Calcium Channel: Predicting Pathogenic/Likely Pathogenic Status for Variants of Uncertain Clinical Significance

**DOI:** 10.3390/membranes11080599

**Published:** 2021-08-07

**Authors:** Svetlana I. Tarnovskaya, Anna A. Kostareva, Boris S. Zhorov

**Affiliations:** 1 Almazov National Medical Research Centre, 197341 St. Petersburg, Russia; anna.kostareva@ki.se; 2 St. Petersburg Academic University, Russian Academy of Sciences, 194021 St. Petersburg, Russia; 3 Department of Women’s and Children’s Health and Center for Molecular Medicine, Karolinska Institute, 17177 Stockholm, Sweden; 4 Sechenov Institute of Evolutionary Physiology & Biochemistry, Russian Academy of Sciences, 194223 St. Petersburg, Russia; 5 Department of Biochemistry and Biomedical Sciences, McMaster University, Hamilton, ON L8S 4K1, Canada

**Keywords:** sequence analysis, variant annotation, protein structure, disease informatics, paralogue, missense variants

## Abstract

(1) Background: Defects in gene CACNA1C, which encodes the pore-forming subunit of the human Cav1.2 channel (hCav1.2), are associated with cardiac disorders such as atrial fibrillation, long QT syndrome, conduction disorders, cardiomyopathies, and congenital heart defects. Clinical manifestations are known only for 12% of CACNA1C missense variants, which are listed in public databases. Bioinformatics approaches can be used to predict the pathogenic/likely pathogenic status for variants of uncertain clinical significance. Choosing a bioinformatics tool and pathogenicity threshold that are optimal for specific protein families increases the reliability of such predictions. (2) Methods and Results: We used databases ClinVar, Humsavar, gnomAD, and Ensembl to compose a dataset of pathogenic/likely pathogenic and benign variants of hCav1.2 and its 20 paralogues: voltage-gated sodium and calcium channels. We further tested the performance of sixteen in silico tools in predicting pathogenic variants. ClinPred demonstrated the best performance, followed by REVEL and MCap. In the subset of 309 uncharacterized variants of hCav1.2, ClinPred predicted the pathogenicity for 188 variants. Among these, 36 variants were also categorized as pathogenic/likely pathogenic in at least one paralogue of hCav1.2. (3) Conclusions: The bioinformatics tool ClinPred and the paralogue annotation method consensually predicted the pathogenic/likely pathogenic status for 36 uncharacterized variants of hCav1.2. An analogous approach can be used to classify missense variants of other calcium channels and novel variants of hCav1.2.

## 1. Introduction

L-type Cav1.2 calcium channels are expressed in various excitable cells including cardiomyocytes [1]. Defects in gene CACNA1C, which encodes the pore-forming α1 subunit of the hCav1.2 channel, underlie cardiac disorders such as atrial fibrillation, long QT syndrome, conduction disorders, cardiomyopathies, and congenital heart defects [2]. With the advent of whole-exome sequencing data, public databases are rapidly replenished with new gene variants. Over 350 CACNA1C missense variants are listed in public databases but clinical significance is known for only 12% of the variants. The guideline of the American College of Medical Genetics and Genomics and the Association for Molecular Pathology (ACMG/AMP) recommends the employment of computational tools to predict damaging variants [3]. Numerous computational tools, which are based on different principles, have been developed to predict the pathogenicity and tolerance of genetic variants [4]. The success rate of these tools varies from 60% to 80% [5]. The ACMG/AMP guideline recommends the employment of multiple software programs for variants’ interpretation because individual programs and underlying algorithms have their own strengths and weaknesses. Therefore, the choice of bioinformatics tools is critical for reliable variant interpretation.

The performance of in silico tools is highly variable and depends on the disease phenotype [6]. For instance, tools MCap, MetaSVM, and MetaLR demonstrated the top performance in predicting the pathogenicity for variants associated with abnormalities in the cardiovascular system [6]. However, some methods yielded many false-positive and false-negative predictions of pathogenicity in individual protein families [7,8,9,10]. For example, MetaSVM, which reportedly has a high accuracy in general [11] and in particular for variants associated with cardiovascular diseases, predicted a deleterious effect for 75% of benign variants of the cardiac sodium channel Nav1.5 [7]. Thus, choosing a tool with a high rate of correct predictions for specific protein families allows to improve predictions [7,12]. 

Many statistical and machine learning tools, which use only protein sequences, have been proposed to predict variant pathogenicity. An alternative approach is the paralogous annotation method [7,13] that is based on analysis of variants in the multiple sequence alignment of functionally and structurally related proteins. The method assumes that if one protein is known to have a damaging mutation in a certain position, analogous mutation in the sequentially matching position of another protein is likely a damaging one. Consensus predictions of pathogenicity by both sequence-based methods and the paralogous annotation within the family of structurally related voltage-gated sodium and calcium channels is expected to provide more reliable pathogenicity prediction for hCav1.2 than individual approaches alone.

A comprehensive predictor assessment requires a benchmark with both positive (pathogenic/likely pathogenic) and negative (benign) variants. Here, we composed a dataset to test the performance of various predictors in identifying known pathogenic/likely pathogenic (P/LP) and benign variants in the families of voltage-gated sodium and calcium channels. We collected common (benign) missense variants from the gnomAD database and P/LP missense variants from three databases that are referred to in the next section. We evaluated the performance of 16 popular prediction tools and identified top-performing tools for the hCav1.2 channel and its paralogs. The best-performing bioinformatics tool, ClinPred, and the paralogue annotation method consensually predicted that 36 hCav1.2 variants of uncertain clinical significance are putative pathogenic/likely pathogenic variants. 

## 2. Methods

### 2.1. Data Collection and Preprocessing

Paralogues of the hCav1.2 channel were previously identified [7]. Sequences of hCav1.2 and its paralogues were obtained from the UniProt database [14]. Pathogenic/likely pathogenic variants of hCav1.2 and its paralogues were collected from three databases: Humsavar (https://www.uniprot.org/docs/humsavar, last visited 26 January 2021), Ensembl Variation [15], and ClinVar [16]. Only ‘disease’ variants were extracted from the databases Ensembl and Humsavar. From the ClinVar database, we selected variants that are characterized as ‘pathogenic’ or ‘likely pathogenic’ and are associated with specific clinical conditions. Benign (neutral) variants along with their minor allele frequencies (AF) were obtained from the population database gnomAD (data release 2.1.1, October 2018) [17]. Variants with AF > 0.0001 were considered as benign [17,18]. Variants of uncertain clinical significance were extracted from ClinVar and Ensembl. All variants were combined into one broad dataset (Table S1). 

### 2.2. hCav1.2 Topology

**Figure 1:**
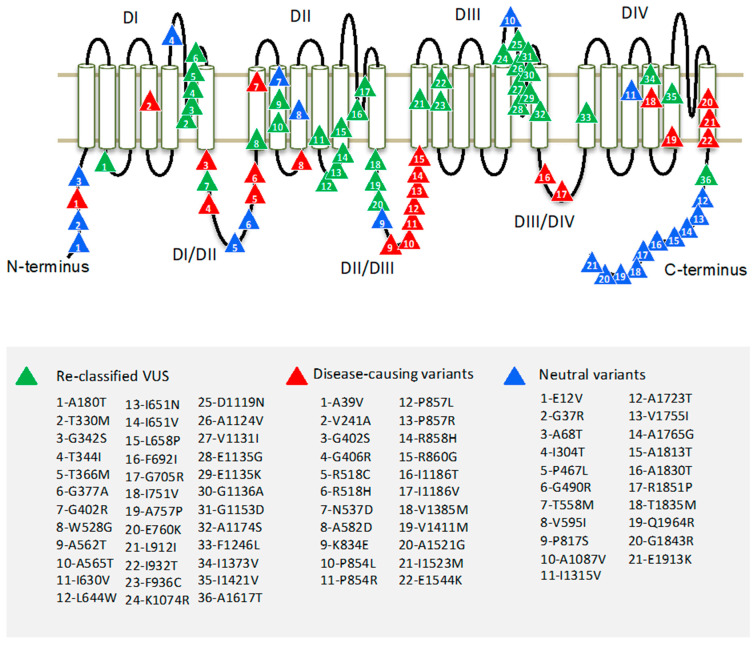
Topology of the hCav1.2 channel. Four repeat domains (DI–DIV) are connected by intracellular linkers. Each domain contains six transmembrane segments (S1–S6). Variants of uncertain clinical significance, which are reclassified here as P/LP variants, are marked by green triangles; known P/LP variants are marked by red triangles; and benign variants are marked by blue triangles.

Region borders of the hCav1.2 channel were determined according to Uniprot entry Q13936. The pore-forming α1 subunit of the Cav1.2 channel folds from a single polypeptide chain of four homologous repeat domains (DI–DIV), which are connected by intracellular linkers. Each domain has six transmembrane helices (S1–S6) and a large extracellular membrane-reentering P-loop (Figure 1). In each repeat, helices S1–S4 constitute a voltage sensing domain, whereas helices S5, S6, and the P-loop contribute a quarter to the pore domain. 

### 2.3. Multiple Sequence Alignment and Paralogue Annotation

The paralogue annotation method specifically identifies P/LP missense variants by transferring annotations across families of related proteins [13,19]. Recently a modified method of paralogue annotation [13] was used to predict the pathogenicity of variants in the cardiac sodium channel hNav1.5 [7]. This approach is applied here to select potential P/LP variants from the large set of VUS in the Cav1.2 channel. 

We have chosen 20 paralogues of hCav1.2: ten voltage-gated sodium channels, nine voltage-gated calcium channels, and a voltage-independent non-selective sodium leak channel (Table 1). For each paralogue, P/LP variants were collected (Section 2.1). Amino acid sequences of Cav1.2 and its paralogues were aligned by Clustal Omega [20]. Each paralogue mutation was mapped on the Cav1.2 sequence according to the alignment (Table S2). Position-specific conservation scores (Cs) were calculated using the Zvelebil method [21] as implemented in the Amino Acid Conservation Calculation Service [22]. Variants in positions with conservation scores > 0.3 were considered as P/LP variants according to References [7,19]. 

### 2.4. Annotation of Missense Variants

We used the dbNSFPv4 database [23] to evaluate the performance of popular tools in predicting P/LP and benign variants. The database contains pre-computed scores for all potential amino acid substitutions that are taken from the following tools: SIFT, Polyphen HVAR, Polyphen HDIV, Mutation Taster, Mutation Assessor, PROVEAN, FATHMM, FATHMM_XF, MetaSVM, MetaLR, CADD, ClinPred, REVEL, PrimateAI, Eigen, and MCap [4]. The performance of algorithms was evaluated using the broad dataset, which includes P/LP and benign variants from hCav1.2 and its 20 paralogues. Variant prioritization tools do not always provide scores for every variant reported in dbNSFP. We selected only those tools in cases where scores are missing for less than 30% of the variants in our dataset. Altogether, 16 different scores were considered. We calculated the area under the ROC (receiver operating characteristic) curve (AUC) using the library pROC in R [24]. The higher the AUC score, the better is the tool performance in the dataset. ROC curves were obtained by plotting sensitivity against (1–specificity) at each threshold for each tool. 

To increase the reliability of the analysis, we determined the optimal pathogenicity threshold using the R package pROC (Table 2). All variants were divided into two categories (P/LP or benign) according to the calculated thresholds. To evaluate the performance of these tools, we calculated the following standard characteristics:

$$\mathrm{Sensitivity}=\frac{TP}{TP+FN}$$

$$\mathrm{Specificity}=\frac{TN}{TN+FP}$$

$$\mathrm{Accuracy}=\frac{TP+TN}{TP+FP+TN+FN}$$

where *TP* (true positive) is the number of P/LP variants correctly predicted as pathogenic; *FN* (false negative) is the number of P/LP variants incorrectly predicted as benign; *TN* (true negative) is the number of benign variants correctly predicted as benign; and *FP* (false positive) is the number of benign variants incorrectly predicted as pathogenic. 

Custom threshold is the custom pathogenicity threshold that divides variants in two categories: pathogenic or benign. The larger or smaller the score than the threshold, the more likely the variant is damaging. Sensitivity characterizes the number of P/LP variants, which were predicted as P/LP by the tool, while specificity characterizes the number of benign variants, which were predicted as benign by the tool. Accuracy indicates the predictive accuracy of the tool [25].

## 3. Results

### 3.1. Composing a Broad Dataset of Missense Variants for Channel hCav1.2 and Its Paralogues

For the 21 channels listed in Table 1, we collected a total of 7164 missense variants from the databases gnomAD, ClinVar, Uniprot, and Humsavar (Table S1). These include 1549 P/LP variants, 763 benign variants (with AF > 0.0001), and 4852 uncharacterized variants or VUS. We further refer to this dataset as the “broad dataset”. The largest numbers of P/LP variants were found for channels hNav1.1, hNav1.5, and hNav1.2 (605, 350, and 166, respectively). No P/LP variants were found for channels hCav3.3 and hNav2.1 (Table 1). For channel hCav1.2, we found 22 P/LP variants, 21 benign variants (with AF > 0.0001), and 309 VUS (Table 1 and Table S1).

### 3.2. Distribution of Missense Variants in Topological Regions of hCav1.2

To identify hCav1.2 regions with pathogenic or benign variants, we explored the occurrence of mutation in the regions. About 70% of hCav1.2 missense variants are localized in cytoplasmic linkers DI-DII and DII-DIII, and in N and C-terminal parts. Most of the P/LP variants appear in linkers DI-DII and DII-DII. Most of the benign variants are localized in the C-terminal part of the channel protein (Figure 1). Highly conserved transmembrane domains contain relatively few P/LP variants.

Most of the P/LP variants of hCav1.2 are associated with long QT syndrome (56%). Other variants are associated with Timothy syndrome, Brugada syndrome, inborn genetic diseases, and other CACNA1C-related disorders (Table S1). Variants causing long QT syndrome and Timothy syndrome most often are localized in interdomain linkers DI-DII and DII-DIII. Some variants are associated with two or more diseases. 

### 3.3. Amino Acid Substitutions in P/LP and Benign Variants

We analyzed statistics of amino acid substitutions in the 21 channels. Arg and Leu have high mutation rates in the P/LP variants, whereas Arg and Ala are highly mutable in benign variants. The most frequent event, irrespective of disease relevance, is the substitution of a hydrophobic residue by another hydrophobic residue. Hydrophobic substitutions of Leu by Pro are frequently associated with diseases, whereas in neutral variants, substitutions of Pro by Leu are most frequent (Table 3). 

### 3.4. Paralogue Annotation of Variants Identified in hCav1.2

The human genome has 20 paralogues of the Cav1.2 channel: ten voltage-gated sodium channels, nine voltage-gated calcium channels, and a voltage-independent non-selective sodium leak channel (Table 1). Using the multiple sequence alignment of hCav1.2 and its paralogues (Section 2.3), we mapped each paralogue protein residue with a known P/LP variant onto the sequentially matching amino acid of hCav1.2 (Table S2). 

A total of 146 known P/LP variants in paralogues were mapped to 89 variants in Cav1.2 (Table S2). Among these, 11 variants correspond to known P/LP variants of Cav1.2, two correspond to benign variants, and 76 correspond to VUS. Most of the paralogue variants were mapped to linkers DI-DII and DII-DIII, N and C-terminal parts, and S5/S6 loops of repeat domains DI and DIII (Table S2).

### 3.5. Comparing Performance of the Computational Tools 

**Figure 2:**
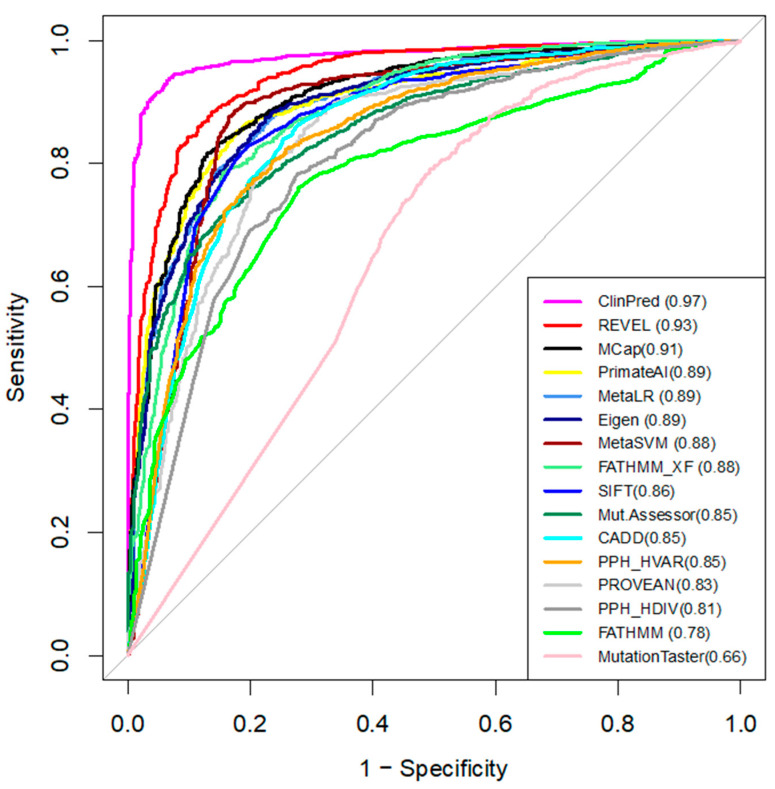
The ROC curves of the 16 tools tested on the broad dataset. The higher AUC score (shown in brackets), the better the performance.

We compared the performance of 16 in-silico tools that predict the pathogenicity of variants (Table 2) [23]. Pre-computed algorithm scores were retrieved from the dbNSFPv4 database. ROC curves and AUC are shown in Figure 2. For each tool, we determined the optimal pathogenicity threshold and calculated sensitivity, specificity, and accuracy (Table 2).

We used AUC as the main measure of performance. ClinPred demonstrated the best predictive performance (AUC = 0.97) in the broad dataset (Figure 2), followed by REVEL (AUC = 0.93) and MCap (AUC = 0.91). Moreover, ClinPred is the only tool that performed with accuracy as (AUC) > 0.90. It correctly classified 95% of the P/LP variants as pathogenic variants and 94% of the benign variants as benign variants (Table 2). MutationAssessor, Polyphen, FATHMM, and MutationTaster performed with an accuracy of < 0.80. The accuracy of other algorithms ranged from 0.80 to 0.88. The lowest accuracy across all methods was found for MutationTaster (accuracy = 0.62, AUC = 0.66). For the hCav1.2 channel, ClinPred, CADD, MetaSVM, Polyphen HDIV, and MutationTaster correctly classified all 22 P/LP variants as pathogenic. However, these methods also assigned pathogenic status for some benign variants. ClinPred predicted 4/21 (19%) benign variants as pathogenic. Polyphen HDIV and MutationTaster classified less than 60% of benign variants as benign. 

The results indicate that ClinPred is the best-performing pathogenicity predictor for variants in the family of voltage-gated sodium and calcium channels.

### 3.6. Reclassifying VUS Variants of hCav1.2 with ClinPred and Paralogue Annotation

Most of the Cav1.2 variants in our broad dataset are currently classified as VUS. We used the best-performing tool, ClinPred, to predict the pathogenicity of VUS’. ClinPred predicted 188 VUS variants as pathogenic/likely pathogenic (pathogenicity threshold > 0.66). Among the 188 VUS, we further selected only those variants that are annotated as pathogenic or likely pathogenic in at least one of 20 paralogs of Cav1.2 (conservation score across paralogues Cs > 0.3). Both methods consensually predicted 36 of 309 VUS as P/LP variants. We reclassified these as putative P/LP variants (Table 4). The variants were localized mainly in the extracellular loops DI-S5/S6 and DIII-S5/S6 (Figure 1). 

## 4. Discussion

Numerous bioinformatics methods are used to predict the deleteriousness of missense variants. These methods rely on supervised machine learning models that are trained on a collection of manually annotated variants to predict the probable pathogenicity for each amino acid substitution. Our recent analysis revealed that in the cardiac sodium channel hNav1.5, the MetaSVM method, which reportedly has a high accuracy in general [11] and in particular for variants associated with cardiovascular diseases [6], predicted a deleterious effect for 75% of variants that are annotated as benign [7]. It is recommended to find a specific predictor with the optimal threshold value for each family of proteins [12].

In the present study, we have shown that various popular bioinformatics tools yield different predictions of pathogenicity for known P/LP and benign variants in the family of the hCav1.2 channel and its paralogues. In our broad dataset, ClinPred performed best with an area of 0.97 under the receiver operating characteristic curve. ClinPred is a meta-predictor that combines commonly used and recently developed individual prediction tool scores, as well as the allele frequency of variants in different populations from the gnomAD database [26]. Its high AUC and prediction accuracy values in our dataset suggest that ClinPred was trained on large newer datasets, which overlap with the dataset used in this study.

## 5. Conclusions

Here, we created a broad dataset that includes all known missense variants in the hCav1.2 channel and 20 paralogues voltage-gated calcium and sodium channels. Most of the known pathogenic/likely pathogenic variants are found in intracellular linkers DI-DII and DII-DIII. Among sixteen pathogenicity prediction tools, which were tested using our broad dataset, ClinPred demonstrated the best performance in distinguishing pathogenic/likely pathogenic variants from benign ones. The best-performing tool is expected to improve in silico assessment of clinically relevant variants of hCav1.2 and its paralogues. ClinPred and the paralogue annotation method consensually predicted that 36 variants of hCav1.2, which are currently classified as variants of unknown significance, are pathogenic/likely pathogenic variants. Most of these variants are located in the extracellular loops DI-S5/S6 and DIII-S5/S6. The reclassified variants can be used for diagnostics of cardiac diseases. They are also promising targets for further experimental and theoretical studies.

## Figures and Tables

**Table 1:** Number of missense variants in the human Cav1.2 and its paralogs.

Gene	Channel	Benign	P/LP	VUS
CACNA1A	hCav2.1	5	60	346
CACNA1B	hCav2.2	48	0	10
CACNA1C	hCav1.2	21	22	309
CACNA1D	hCav1.3	4	7	46
CACNA1E	hCav2.3	36	17	18
CACNA1F	hCav1.4	39	28	39
CACNA1G	hCav3.1	42	5	45
CACNA1H	hCav3.2	116	21	454
CACNA1I	hCav3.3	61	0	1
CACNA1S	hCav1.1	57	11	290
NALCN	hNavi2.1	18	31	17
SCN1A	hNav1.1	18	605	482
SCN2A	hNav1.2	15	166	301
SCN3A	hNav1.3	16	8	202
SCN4A	hNav1.4	47	78	312
SCN5A	hNav1.5	43	350	705
SCN7A	hNav2.1	65	0	0
SCN8A	hNav1.6	6	93	249
SCN9A	hNav1.7	11	32	505
SCN10A	hNav1.8	66	3	291
SCN11A	hNav1.9	29	12	230

**Table 2:** Performance of variant interpretation tools.

Tool	Custom Threshold	Sensitivity	Specificity	Accuracy
ClinPred	>0.66	0.95	0.94	0.95
REVEL	>0.73	0.89	0.87	0.88
MetaLR	>0.91	0.86	0.84	0.85
Eigen	>0.55	0.81	0.87	0.84
MCap	>0.59	0.82	0.87	0.84
PrimateAI	>0.69	0.88	0.79	0.84
MetaSVM	>0.92	0.88	0.85	0.87
FATHMM_XF	>0.85	0.82	0.84	0.83
SIFT	<0.0025	0.83	0.83	0.83
PROVEAN	<−3.42	0.80	0.80	0.80
CADD	>24	0.88	0.72	0.80
MutationAssessor	>2.51	0.77	0.81	0.79
Polyphen HVAR	>0.6	0.80	0.70	0.75
FATHMM	<−4.305	0.76	0.73	0.74
Polyphen HDIV	>0.96	0.74	0.71	0.73
MutationTaster	>0.99	0.94	0.31	0.62

**Table 3:** Occurrence of top five residue types in P/LP and benign variants.

Type of Data	WT Residue	Mutant Residue	Preferred Substitutions
All variants (*n* = 2312)	R, A, V, L, G	V, T, S, R, I	R > Q, L > P, R > H, A > T, R > C
P/LP variants (*n* = 1549)	R, L, V, G, A	M, R, C, D, H	L > P, G > R, R > Q, R > H, R > C
Benign variants (*n* = 763)	R, A, P, V, G	V, S, T, A, R	A > T, R > Q, P > L, R > H, V > I

**Table 4:** hCav1.2 variants whose status was changed from “uncertain significance” to “pathogenic/likely pathogenic”.

#	Variant	Location	Paralogue
1	A180T	DI-S2/S3	SCN5A-A178G, SCN1A-A175V, SCN1A-A175T
2	T330M	DI-S5/S6	SCN1A-Y349C
3	G342S	DI-S5/S6	SCN5A-G351V, SCN5A-G351D
4	T344I	DI-S5/S6	SCN1A-T363R, SCN1A-T363P, SCN5A-T353I
5	T366M	DI-S5/S6	SCN1A-E385Q
6	G377A	DI-S5/S6	SCN5A-G386R, SCN5A-G386E
7	G402R	DI-DII	CACNA1E-G348R, SCN1A-A420V, CACNA1D-G403D, CACNA1F-G369D
8	W528G	DII-S1	SCN1A-L772P
9	A562T	DII-S2	CACNA1H-V831M
10	A565T	DII-S2	SCN5A-G758E
11	I630V	DII-S4	SCN1A-L869S, SCN1A-L869F
12	L644W	DII-S4/S5	SCN9A-I859T, SCN3A-I875T, SCN4A-I693T, SCN8A-I868T
13	I651N	DII-S4/S5	SCN5A-L839P, CACNA1E-I603L, SCN8A-L875Q, CACNA1A-I614M, SCN1A-L890P
14	I651V	DII-S4/S5	SCN5A-L839P, CACNA1E-I603L, SCN8A-L875Q, CACNA1A-I614M, SCN1A-L890P
15	L658P	DII-S5	NALCN-T513N, SCN1A-L897S, SCN1A-L897F
16	F692I	DII-S5/S6	SCN2A-F928C
17	G705R	DII-S5/S6	SCN1A-G950E, SCN1A-G950R
18	I751V	DII-DIII	CACNA1D-I750F, CACNA1A-I712V, CACNA1E-I701V, CACNA1F-I756T, CACNA1D-I750M
19	A757P	DII-DIII	SCN2A-S987I
20	E760K	DII-DIII	SCN1A-D998G
21	L912I	DIII-S1	SCN1A-L1230F
22	I932T	DIII-S2	SCN1A-M1251R, SCN5A-L1238P
23	F936C	DIII-S2	SCN1A-A1255D, SCN1A-A1255P
24	K1074R	DIII-S5/S6	SCN5A-K1359N
25	D1119N	DIII-S5/S6	SCN1A-D1416G
26	A1124V	DIII-S5/S6	SCN1A-G1421R, SCN1A-G1421E, SCN5A-G1408R
27	V1131I	DIII-S5/S6	CACNA1A-V1456L, SCN1A-V1428A, SCN1A-V1428F
28	E1135G	DIII-S5/S6	SCN1A-K1432I, SCN5A-K1419E, SCN2A-K1422E
29	E1135K	DIII-S5/S6	SCN1A-K1432I, SCN5A-K1419E, SCN2A-K1422E
30	G1136A	DIII-S5/S6	SCN1A-G1433V, SCN5A-G1420R, SCN5A-G1420V, SCN1A-G1433R, SCN1A-G1433E
31	G1153D	DIII-S5/S6	SCN1A-Q1450K, SCN1A-Q1450R
32	A1174S	DIII-S6	SCN5A-S1458Y, SCN1A-S1471F
33	F1246L	DIV-S1	SCN4A-M1360V, SCN2A-M1538I
34	I1373V	DIV-S4	SCN1A-P1632S
35	I1421V	DIV-S5	SCN1A-I1683F, SCN4A-I1495F, SCN1A-I1683T
36	A1617T	C-term	SCN5A-V1861I

## Supplementary Materials

The following are available online at https://www.mdpi.com/article/10.3390/membranes11080599/s1. Table S1: Broad dataset of Cav1.2 variants and its paralogues, and Table S2: Mapping of P/LP paralogue variants on the human Cav1.2 sequence.

## Abbreviations

AF, allele frequencies; AUC, area under the ROC curve; DI-DIV, repeat domains in eukaryotic sodium and calcium channels; P/LP, pathogenic/likely pathogenic variants; ROC, receiver operating characteristic; S1-S6, transmembrane segments; S1/S2, S3/S4, and S5/S6, extracellular loops between transmembrane segments; S2/S3, cytoplasmic loops between transmembrane segments; S4/S5, linker helices between voltage-sensing domains and the pore domain; and VUS, variant of uncertain significance.
